# Large Cyst of Skene Gland: A Rare Perineum Mass

**DOI:** 10.1055/s-0043-1768944

**Published:** 2023-05-14

**Authors:** Konstantinos Tzelepis, Konstantina Zacharouli, Athina A. Samara, Antonios Koutras, Emmanuel N. Kontomanolis, Konstantinos Perivoliotis, Efterpi Pavlidou, Sotirios Sotiriou

**Affiliations:** 1 Department of Urology, General Hospital of Nicaea-Piraeus, Pireas, Greece; 2 Department of Pathology, Faculty of Medicine, School of Health Sciences, University of Thessaly, Larissa, Greece; 3 Laboratory of Histology and Embryology, Faculty of Medicine, School of Health Sciences, University of Thessaly, Larissa, Greece; 4 Department of Obstetrics and Gynecology, Alexandra Hospital, Kapodistrian University of Athens, Athens, Greece; 5 Department of Obstetrics and Gynecology, Faculty of Medicine, School of Health Sciences, Democritus University of Thrace, Alexandroupolis, Greece; 6 Department of Surgery, General Hospital of Volos, Volos, Greece; 7 Department of Speech and Language Therapy, University of Ioannina, Ioannina, Greece

**Keywords:** cyst, Skene's gland, paraurethral gland, marsupialization

## Abstract

**Objective** In this report we present a rare case of a large cyst of Skene gland in a female patient with a palpable vaginal mass persisting for at least 2 years.

**Case Report** A 67-year-old female admitted to the department of urology due to the presence of “a vaginal mass” for the past 2 years. A cyst of Skene's duct was suspected based on clinical manifestation and findings of magnetic resonance imaging showing an extensive cyst formation in the upper vaginal area and anterior to the urethra. Based on these findings, a decision for surgical removement of the cyst was made. The cyst was incised, drained, and marsupialized. The postoperative recovery was uneventful, and the patient was discharged on the second postoperative day.

**Conclusion** High clinical suspicion is important to reach this rare diagnosis. Partial excision and marsupialization of the cyst is a simple procedure with low morbidity, without recurrence, and excellent results.

Dutch histologist Reinier de Graaf was the first to describe the presence of the female prostate formed by the glands and ducts located around the female urethra. 1 Two hundred years later gynecologist Alexander J.C. Skene described the glands bearing his name as consisting of two main paraurethral ducts that open on the sides of the urethral orifice. 1 Skene's glands or paraurethral glands are located around the lower end of the female urethra. 1 Their function consists in producing, after sexual stimulation, a mucoid secretion (female ejaculation) which protects and lubricates the urethral opening. 2

These glands are homologous to the male prostate that are developed from the same embryological tissues, being associated with various pathologies. 1 Interestingly, in 1997 Cabello, reported different prostate-specific antigen (PSA) values in urine samples from women before and after orgasm. 1 These findings can be explained by the function of Skene's glands secreting fluid containing PSA, acid phosphatase, and high concentrations of glucose and fructose. 3 The function of the female prostatic tissue is not entirely clear. O'Connell et al suggests that the distal urethra, vagina, and clitoris have common vascularity and innervation and constitute a tissue mass that is related to normal sexual function. 4

Infections, cystic and solid, benign, or malignant tumors are typical disorders of these glands. 1 For tumors of Skene glands, surgical treatment remains the treatment of choice, to manage common symptoms of these diseases such as urinary incontinence, vaginal prolapse, or urethral and paraurethral pathologies that may interfere with female sexual function. 1 2 3 4

In the present report, we aim to present an interesting case of a female patient with a large and symptomatic Skene's gland cyst, underlining the importance of high clinical suspicion to reach the rare diagnosis and present the current literature.

## Case Presentation

A 67-year-old female admitted to the department of urology due to the presence of “a vaginal mass” for the past 2 years. Her symptoms included dyspareunia, frequency and urgency of urination, interruption, and distortion of urinary flow as well as episodes of vaginal bleeding. Her past medical history was insignificant.

**Fig. 1:**
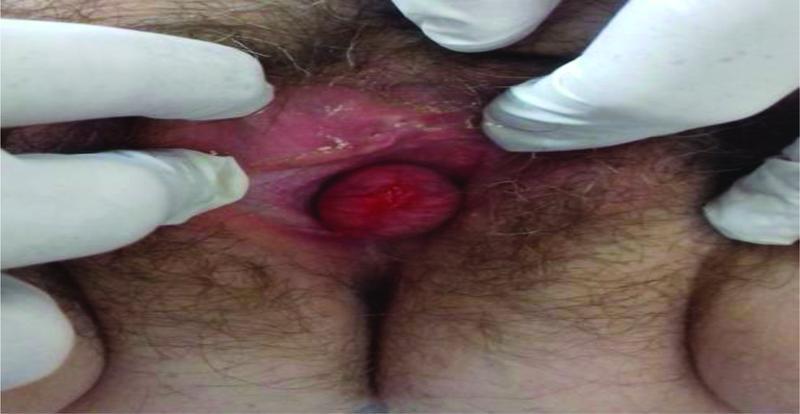
Large gland Skene cyst.

**Fig. 2:**
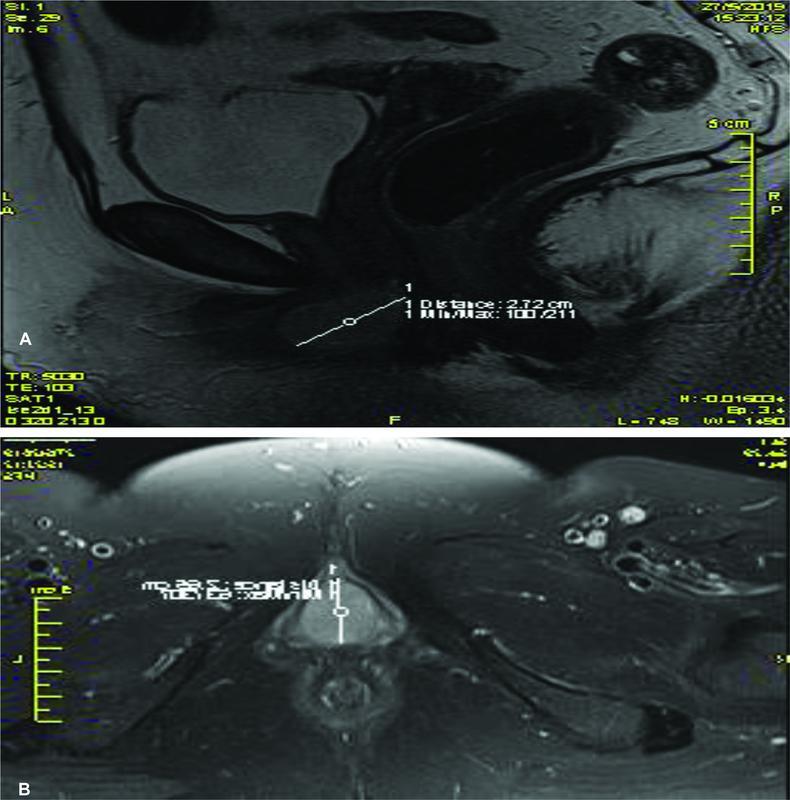
( **A** , **B** ) Magnetic resonance imaging (MRI) imaging of cyst gland Skene.

On vaginal examination, a cystic mass of 5 cm in diameter with ulceration covering the urethral meatus was observed ( Fig. 1 ). A cyst of Skene's duct was suspected based on clinical manifestation and findings of magnetic resonance imaging (MRI) showing an extensive cyst formation in the upper vaginal area and anterior to the urethra ( Fig. 2A, B ). An urethrocystoscopy without any findings was performed to exclude other pathologies.

Based on these findings, a decision for surgical removement of the cyst was made. The cyst was incised, drained, and marsupialized with interrupted 4–0 Vicryl sutures. The excised tissue was sent for histological examination while the posterior wall of the cyst remained in place avoiding the complete excision of the cyst to minimize the risk of urethral lesions. The postoperative recovery was uneventful, and the patient was discharged on the second postoperative day.

The histopathology examination confirmed the diagnosis by reporting a cyst covered with stratified squamous epithelium. Patient remained asymptomatic, with no signs of recurrence during the next 6 months.

## Discussion

Most common pathologies associated with Skene's glands are infections, such as gonorrhea, tuberculosis, and trichomoniasis, and can be present both in the glands and other parts of the reproductive system. 5 Solid or cystic benign tumors (simple cysts, prostate antigen positive vaginal polyps) represent another related pathology and rare malignant tumors such as urethral adenocarcinoma which can induce an increase in PSA up to 5.9 ng/mL have been reported in the literature. 1 6

Obstruction of the ducts of these glands leads to the formation of cysts that present as benign cystic masses inferolateral to the urethral meatus, typically affecting in women in the third or fourth decade of their life. 7 However, Skene's gland cyst has been observed in both female neonates and preadolescent girls. 8 Skene's gland cysts may be presented in newborns as a congenital anomaly, where cystic degeneration of embryonic remnants of the paraurethral glands has been considered as one of the causes. In female neonates, paraurethral cysts generally appear as an asymptomatic, small protruding yellow or whitish mass with superficial small vessels, located on either side of the urethral meatus. The location and the displacement of the urethral meatus by the mass are characteristic of paraurethral cysts. 9 On the other hand, in adolescence-onset cases, the reasons some cysts are increasing in measure remain unclear. Most cases are presumably related to partial obstruction mainly due to infection. 8

According to Lucioni et al, Skene's gland is thought to be the female homologue of the male prostate. 10 In males, prostate cancer and benign prostatic hyperplasia, are related to male hormones. Based on our search in the current literature, there are no reports between direct relationship of Skene's gland cysts and such hormones. Recently though, Skene's adenocarcinoma, a very rare entity derived from Skene's gland, and its similarity with prostate cancer has been reported, describing the poorly formed glands of pattern 4 and positive staining for NKX3.1. 11 According to Pollen and Dreilinger and Tepper et al, Skene's glands not only histologically showed resemblance to prostate cancer but were expressing immunohistochemical staining for PSA. 11 12 In 2018, Tregnago and Epstein 13 presented the first series of four cases and certified that Skene's gland adenocarcinoma has similar microscopical features to high-grade prostatic adenocarcinoma with a cribriform predominant pattern as well as histology analogous to Gleason pattern 3 and solid sheets of cells correspond to Gleason pattern 5 staining for PSA.

Since Skene's ducts arise from the urogenital sinus, these cysts are usually lined with stratified squamous epithelium. 14 Symptoms include a visible or palpable interlabial vaginal mass, presenting with pain, dyspareunia, urinary disturbances, abscess formation, or may be completely asymptomatic discovered accidentally during a routine vaginal exam. Etiological factors of Skene's gland cysts include infections or mechanical trauma while the most important differential diagnosis is the urethral diverticulum which is usually located on the middle urethra. 15

The history, physical examination, and urethrocystoscopy are often sufficient to set the diagnosis of the simple cyst or Skene's gland abscess. Transperineal ultrasound and MRI can be useful tools for a more detailed examination with the use of MRI as the gold standard examination of female urethra. 14 16

When conservative treatments fail, surgical options include needle aspiration, partial excision, marsupialization, or complete excision of the cyst. 17 Partial excision of the cyst and marsupialization leaving the posterior wall of the cyst in place is considered an effective treatment avoiding the risk of further urethral lesions that can otherwise occur in an attempt of complete excision. However, despite reports of complete excision and marsupialization, to our knowledge there are no comparative studies evaluating outcomes of the two techniques. 18 Shah et al 19 in a case series of 34 patients, reported that after complete surgical excision, 30% of patients needed further treatment, with 85.3% overall success rate of surgical treatment after all treatments. Furthermore, two case series with a patient population of 10 women each, reported no recurrences of Skene's gland cyst excision at a mean follow-up of 46 and 8 months, respectively. 8 20 On the other hand, Sharifi-Aghdas and Ghaderian ^21^ in a case series of 25 patients, found no recurrence of Skene's gland cysts after partial excision with marsupialization.

## Conclusion

Large Skene's cysts are very rare benign lesions. History, clinical examination, and endoscopy are often sufficient for a definitive diagnosis. High clinical suspicion is important to reach this rare diagnosis. Partial excision and marsupialization of the cyst is a simple procedure with low morbidity, no recurrence, and excellent results.

## References

[1] Zaviacic M Ablin R J The female prostate and prostate-specific antigen. Immunohistochemical localization, implications of this prostate marker in women and reasons for using the term “prostate” in the human female(Review)Histol Histopathol 2000 15 01 131 142 10668204 10.14670/HH-15.131

[2] Rodriguez F D Camacho A Bordes S J Gardner B Levin R J Tubbs R S Female ejaculation: an update on anatomy, history, and controversies(Review)Clin Anat 2021 34 01 103 107 32681804 10.1002/ca.23654

[3] Pastor Z Chmel R Differential diagnostics of female “sexual” fluids: a narrative review(Review)Int Urogynecol J Pelvic Floor Dysfunct 2018 29 05 621 629 10.1007/s00192-017-3527-9 29285596

[4] O'Connell H E Hutson J M Anderson C R Plenter R J Anatomical relationship between urethra and clitoris J Urol 1998 159 06 1892 1897 9598482 10.1016/S0022-5347(01)63188-4

[5] Petrin D Delgaty K Bhatt R Garber G Clinical and microbiological aspects of Trichomonas vaginalis(Review)Clin Microbiol Rev 1998 11 02 300 317 9564565 10.1128/cmr.11.2.300 PMC106834

[6] Stewart C J Tubulo-squamous vaginal polyp with basaloid epithelial differentiation Int J Gynecol Pathol 2009 28 06 563 566 19851205 10.1097/PGP.0b013e3181a9cd86

[7] Nickles S W Burgis J T Menon S Bacon J L Prepubertal Skene's abscess J Pediatr Adolesc Gynecol 2009 22 01 e21 e22 10.1016/j.jpag.2007.11.004 19232290

[8] Foster J Lemack G Zimmern P Skene's gland cyst excision Int Urogynecol J Pelvic Floor Dysfunct 2016 27 05 817 820 10.1007/s00192-015-2872-9 26670578

[9] Kusama Y Ito K Suzuki T Skene's duct cyst J Gen Fam Med 2017 18 05 299 300 29264050 10.1002/jgf2.64 PMC5689431

[10] Lucioni A Rapp D E Gong E M Fedunok P Bales G T Diagnosis and management of periurethral cysts Urol Int 2007 78 02 121 125 17293650 10.1159/000098068

[11] Pollen J J Dreilinger A Immunohistochemical identification of prostatic acid phosphatase and prostate specific antigen in female periurethral glands Urology 1984 23 03 303 304 6199882

[12] Tepper S L Jagirdar J Heath D Geller S A Homology between the female paraurethral (Skene's) glands and the prostate. Immunohistochemical demonstration Arch Pathol Lab Med 1984 108 05 423 425 6546868

[13] Tregnago A C Epstein J I Skene's glands adenocarcinoma: a series of 4 cases Am J Surg Pathol 2018 42 11 1513 1521 29901570 10.1097/PAS.0000000000001108

[14] Chaudhari V V Patel M K Douek M Raman S S MR imaging and US of female urethral and periurethral disease Radiographics 2010 30 07 1857 1874 21057124 10.1148/rg.307105054

[15] Tamburrini S Vascone C Marrone V Skene's glands abscess an overlooked diagnosis in acute lower urinary symptoms Radiol Case Rep 2021 16 12 3751 3756 34630812 10.1016/j.radcr.2021.09.006 PMC8493500

[16] Maetzold E Takacs E B Urethral Pathology in Women Curr Urol Rep 2022 23 10 225 234 36114996 10.1007/s11934-022-01109-6

[17] Sagar R Shah M D Victor W Nitti M Benign vaginal wall masses and paraurethral lesions 1st ed. Philadelphia Elsevier Saunders 2012 127 135

[18] Laura M Neeraja C Denise B Lisa C Willy D G Skene's gland cyst: a simple marsupialization technique Int Urogynecol J Pelvic Floor Dysfunct 2017 28 07 1101 1102 10.1007/s00192-016-3246-7 28032188

[19] Shah S R Biggs G Y Rosenblum N Nitti V W Surgical management of Skene's gland abscess/infection: a contemporary series Int Urogynecol J Pelvic Floor Dysfunct 2012 23 02 159 164 10.1007/s00192-011-1488-y 21732101

[20] Köse O Aydemir H Metin O Budak S Sonbahar A Adsan O Experiences with the management of paraurethral cysts in adult women Cent European J Urol 2014 66 04 477 480 10.5173/ceju.2013.04.art24 PMC3992454 24757549

[21] Sharifi-Aghdas F Ghaderian N Female paraurethral cysts: experience of 25 cases BJU Int 2004 93 03 353 253 10.1111/j.1464-410x.2003.04615.x. PMID: 14764136 14764136

